# Anxiety and depression during the first wave of COVID-19 in Germany – results of repeated cross-sectional surveys

**DOI:** 10.1017/S0033291721000866

**Published:** 2021-03-02

**Authors:** A.-K. Bräscher, C. Benke, B. M. Weismüller, E. Asselmann, E.-M. Skoda, M. Teufel, S. M. Jungmann, M. Witthöft, C. A. Pané-Farré

**Affiliations:** 1 Department for Clinical Psychology, Psychotherapy, and Experimental Psychopathology, Johannes Gutenberg-University Mainz, Germany; 2 Department of Psychology, Clinical Psychology, Experimental Psychopathology, and Psychotherapy, University of Marburg, Marburg, Germany; 3 LVR University Hospital, Clinic for Psychosomatic Medicine and Psychotherapy, University of Duisburg-Essen, Essen, Germany; 4 Department of Psychology, Personality Psychology, Humboldt University of Berlin, Berlin, Germany

Numerous studies have reported elevated psychological distress during the coronavirus disease 2019 (COVID-19) pandemic in Germany (Bäuerle et al., 2020; Benke, Autenrieth, Asselmann, & Pané-Farré, 2020; Jungmann & Witthöft, 2020) and other countries worldwide (Prati & Mancini, 2021). However, longitudinal or repeated cross-sectional studies investigating the role of lockdown restrictions on mental health are rare although such studies promise to be highly useful to identify persons at risk to develop increased anxiety and depression (Daly, Sutin, & Robinson, 2020; Debowska, Horeczy, Boduszek, & Dolinski, 2020). This study aimed at examining whether symptoms of depression and anxiety were increased during *v.* before the first COVID-19 lockdown in Germany, whether symptoms remained elevated after lockdown restrictions were eased, and whether the observed effects were predicted by sociodemographic variables.

Three cross-sectional online surveys in Germany (Fig. 1) assessed anxiety (GAD-2; Spitzer, Kroenke, Williams, & Löwe, 2006), depression (PHQ-2; Kroenke, Spitzer, & Williams, 2001), and sociodemographic factors during the *early stage* of the first COVID-19 wave, i.e. prior to the lockdown (10th March – 24th of March 2020; *N* = 11 220), at *mid-stage*, i.e. during peak of daily infections and lockdown-related restrictions (25th March – 13th April 2020, *N* = 4268), and during *late stage*, i.e. ease of lockdown and decline of daily infection rates (17th April–15th May 2020, *N* = 4335). All three samples were recruited via convenience sampling methods and differed regarding sex (early stage: 72.2% females; mid-stage: 79.2% females; late stage: 75.8% females; χ^2^(1) = 83.94, *p* < 0.001), age (early stage: 39.2% 16–34 years, 42.0% 35–54 years, 18.8% 55 and older; mid-stage: 65.4% 16–34 years, 26.9% 35–54 years, 7.7% 55 and older; late stage: 49.1% 16–34 years, 35.6% 35–54 years, 15.3% 55 and older; χ^2^(2) = 1095.38, *p* < 0.001), employment status (early stage: 74.4% employed, 9.9% unemployed, 13.0% students/in training, 1.6% retired; mid-stage: 52.6% employed, 5.6% unemployed, 34.9% students/in training, 3.1% retired; late stage: 69.6% employed, 6.6% unemployed, 17.3% students/in training, 6.2% retired; χ^2^(3) = 1342.63, *p* < 0.001), children (early stage: 28.3% having children; mid-stage: 29.5% having children; late stage: 49.1% having children; χ^2^(1) = 641.60, *p* < 0.001), and history of mental disorders (early stage: 12.9% with mental disorder; mid-stage: 13.4% with mental disorder; late stage: 38.0% with mental disorder; χ^2^(1) = 1410.18, *p* < 0.001). The samples did not differ regarding level of education (early stage: 4.5% low, 50.4% medium, 44.5% high; mid-stage: 4.5 low, 51.4% medium, 43.7% high; late stage: 4.2% low, 51.5% medium, 44.2% high; χ^2^(2) = 1.86, *p* < 0.762).

**Fig. 1.**
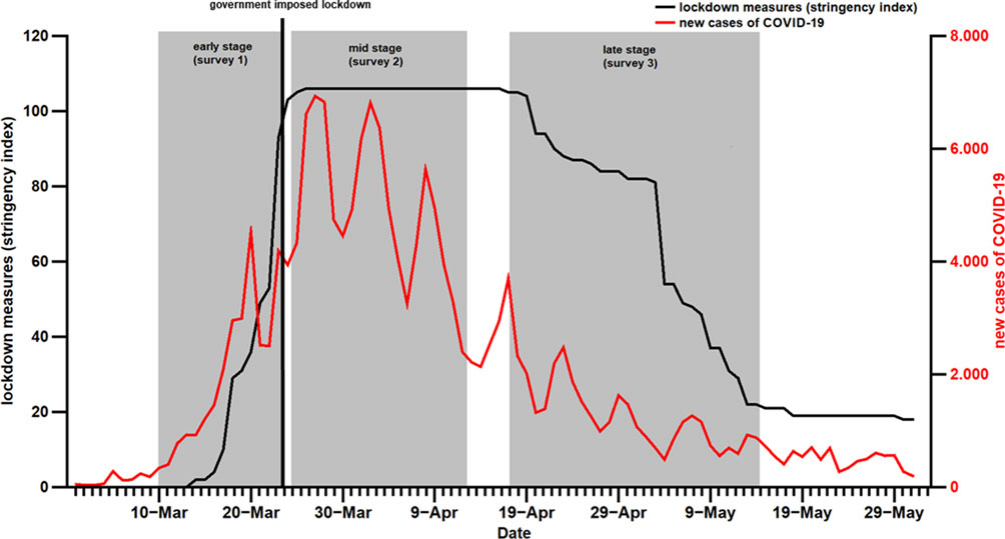
Course of new cases of COVID-19 and restrictions due to lockdown measures (e.g. prohibition to meet with others in public places, closure of non-essential shops, or prohibition to leave the apartment without reason) during the first wave of the pandemic in Germany. The time points and durations of the three surveys are shaded in grey.

Stage (contrast coded: early *v.* mid- and late stage; mid- *v.* late stage) and sociodemographic variables served as predictors in the first step of hierarchical multiple regressions for anxiety and depression, respectively. In a second step, interactions of the contrast-coded variables of stage with sociodemographic variables were entered into the regression.

Overall and in line with our previous findings (Bäuerle et al., 2020; Benke et al., 2020; Jungmann & Witthöft, 2020), women *v.* men, younger *v.* older individuals, individuals with lower *v.* higher educational level, individuals who were unemployed *v.* employed, and with *v.* without a previous history of mental disorders experienced higher anxiety and depressive symptoms during the first COVID-19 wave (see Table 1). Moreover, living without *v.* with children was associated with more depression but not anxiety.

## Anxiety and depression during *v.* before lockdown:

Anxiety and depression were significantly higher during the lockdown than before. However, these symptom differences were greater in younger *v.* older individuals, in less *v.* more educated individuals and in individuals without *v.* with a previous history of mental disorders (see Table 1). Moreover, the symptom difference in anxiety (with higher anxiety during the lockdown *v.* before) was greater in men *v.* women and unemployed *v.* employed individuals.

**Table 1.** Descriptive statistics (*M*, *s.d.*) of sample characteristics and coefficients of multiple hierarchical regression analyses for anxiety and depression

Predictor	*β* (*p* value)	Standard error	*M* (*s.d.*) of stage			Interaction of sociodemographics with contrast 1 (early *v.* mid- and late stage)^b^	Standard error	Interaction of sociodemographics with contrast 2 (mid- *v.* late stage)^c^	Standard error
			Early	Mid	Late				
	**Anxiety**								
**Stage of the first wave of COVID-19**			1.44 (1.62)	1.73 (1.65)	1.69 (1.62)	**0.015 (0.031)**		**0.059 (< 0.001)**	
**Sex**									
Male^a^			1.05 (1.38)	1.34 (1.54)	1.55 (1.65)				
Female	**0.091 (< 0.001)**	0.03	1.59 (1.68)	1.83 (1.67)	1.74 (1.61)	**−0.015 (0.023)**	0.02	**0.025 (< 0.001)**	0.04
**Age**									
16–34 years	**0.102 (< 0.001)**	0.04	1.60 (1.71)	1.71 (1.66)	1.92 (1.73)	**0.036 (0.004)**	0.03	**−0.032 (0.017)**	0.07
35–54 years	**0.078 (< 0.001)**	0.04	1.40 (1.59)	1.86 (1.66)	1.66 (1.58)	**0.028 (0.019)**	0.03	−0.013 (0.293)	0.06
55 years and Older^a^			1.20 (1.44)	1.43 (1.57)	1.29 (1.39)				
**Education**									
Low^a^			1.54 (1.67)	2.16 (1.93)	1.93 (1.89)				
Medium	**−0.037 (0.028)**	0.06	1.51 (1.67)	1.82 (1.71)	1.78 (1.66)	−0.012 (0.467)	0.04	0.006 (0.732)	0.08
High	**−0.061 (< 0.001)**	0.06	1.34 (1.54)	1.57 (1.54)	1.56 (1.53)	**−0.036 (0.033)**	0.04	< 0.001 (0.983)	0.08
**Employment**									
Employed^a^			1.35 (1.56)	1.71 (1.62)	1.57 (1.55)				
Student/in training	**0.023 (0.006)**	0.03	1.76 (1.74)	1.64 (1.65)	1.93 (1.69)	**−0.020 (0.016)**	0.02	**−0.021 (0.013)**	0.05
Unemployed	**0.041 (< 0.001)**	0.04	1.71 (1.81)	2.22 (1.67)	2.29 (1.83)	**0.018 (0.010)**	0.03	−0.012 (0.082)	0.07
Retired	< 0.001 (0.978)	0.08	1.18 (1.40)	1.76 (1.84)	1.73 (1.74)	0.014 (0.088)	0.05	−0.010 (0.204)	0.09
**Children**									
No^a^			1.47 (1.63)	1.67 (1.65)	1.81 (1.71)				
Yes	0.005 (0.486)	0.03	1.36 (1.58)	1.86 (1.67)	1.56 (1.52)	0.010 (0.299)	0.02	**0.027 (0.004)**	0.04
**History of mental disorder**									
No^a^			1.22 (1.44)	1.52 (1.52)	1.29 (1.38)				
Yes	**0.342 (< 0.001)**	0.03	2.93 (1.93)	3.04 (1.85)	2.34 (1.77)	**−0.046 (< 0.001)**	0.02	**0.038 (< 0.001)**	0.04
	**Depression**								
**Stage of the first wave of COVID-19**			1.01 (1.43)	1.93 (1.54)	2.00 (1.60)	**0.245 (< 0.001)**		**0.025 (< 0.001)**	
**Sex**									
Male^a^			0.90 (1.33)	1.78 (1.54)	2.02 (1.68)				
Female	0.009 (0.182)	0.02	1.05 (1.47)	1.97 (1.54)	1.99 (1.57)	0.008 (0.239)	0.02	**0.019 (0.003)**	0.04
**Age**									
16–34 years	**0.133 (< 0.001)**	0.04	1.15 (1.56)	2.03 (1.54)	2.36 (1.68)	**0.083 (< 0.001)**	0.03	−0.016 (0.225)	0.06
35–54 years	**0.089 (< 0.001)**	0.04	0.94 (1.35)	1.84 (1.55)	1.89 (1.52)	**0.041 (< 0.001)**	0.02	0.004 (0.765)	0.06
55 years and older^a^			0.87 (1.30)	1.37 (1.42)	1.51 (1.45)				
**Education**									
Low^a^			1.35 (1.67)	2.53 (1.66)	2.32 (1.76)				
Medium	**−0.084 (< 0.001)**	0.05	1.12 (1.52)	2.06 (1.58)	2.10 (1.61)	−0.021 (0.184)	0.03	−0.011 (0.494)	0.08
High	**−0.136 (< 0.001)**	0.05	0.84 (1.27)	1.71 (1.45)	1.86 (1.56)	**−0.034 (0.035)**	0.03	−0.020 (0.225)	0.08
**Employment**									
Employed^a^			0.90 (1.31)	1.83 (1.52)	1.88 (1.52)				
Student/in training	**0.030 (< 0.001)**	0.03	1.34 (1.70)	2.03 (1.52)	2.36 (1.69)	**−0.017 (0.033)**	0.02	−0.011(0.177)	0.04
Unemployed	**0.041 (< 0.001)**	0.04	1.40 (1.78)	2.19 (1.67)	2.40 (1.79)	−0.002 (0.804)	0.03	−0.010 (0.136)	0.06
Retired	0.009 (0.234)	0.07	0.98 (1.36)	1.79 (1.79)	1.90 (1.76)	0.006 (0.452)	0.05	−0.004 (0.608)	0.08
**Children**									
No^a^			1.08 (1.49)	1.96 (1.53)	2.20 (1.67)				
Yes	**−0.031 (< 0.001)**	0.02	0.83 (1.26)	1.86 (1.57)	1.79 (1.49)	0.010 (0.301)	0.02	0.012 (0.167)	0.04
**History of mental disorder**									
No^a^			0.78 (1.18)	1.77 (1.45)	1.69 (1.47)				
Yes	**0.335 (< 0.001)**	0.03	2.54 (1.95)	2.96 (1.72)	2.50 (1.68)	**−0.092 (< 0.001)**	0.02	**0.029 (< 0.001)**	0.04

a Reference category of dummy-coded variable.

b Contrast-coded stage of the first wave of COVID-19: early (−2), mid- (1), late (1).

c Early (0), mid- (1), late (−1).

## Anxiety and depression during peak of *v.* easing of lockdown restrictions

Overall, depression and anxiety decreased during easing compared to peak of lockdown restrictions (see Table 1). However, anxiety was increased during the easing *v.* peak of restrictions in younger *v.* older individuals, in men *v.* women, in students/individuals in training *v.* employed individuals and in individuals without *v.* with children (see Table 1). Depression during easing *v.* peak of lockdown measures was higher in men *v.* women. The symptom difference in depression (with lower symptoms during easing *v.* peak of lockdown measures) was greater in individuals with *v.* without a history of mental disorders.

The results suggest that implementation of lockdown restrictions is associated with increased levels of depression and anxiety that even persist during ease of lockdown in specific groups. Men and individuals without a history of mental disorders were particularly affected by lockdown restrictions. The results are consistent with previous longitudinal studies from the UK, which demonstrated that younger individuals experienced higher levels of distress due to lockdown measures (Daly et al., 2020; Pierce et al., 2020). This study identifies vulnerable groups (Bäuerle et al., 2020; Benke et al., 2020; Jungmann & Witthöft, 2020), which might need tailored support to avoid exacerbation or the development of manifest psychological disorders. Longitudinal studies based on representative community samples are needed to replicate our findings.

## References

[ref1] Bäuerle, A., Teufel, M., Musche, V., Weismüller, B. M., Kohler, H., Hetkamp, M., … Skoda, E.-M. (2020). Increased generalized anxiety, depression and distress during the COVID-19 pandemic: A cross-sectional study in Germany. Journal of Public Health, 42(4), 672–678. doi:10.1093/pubmed/fdaa106. 32657323 PMC7454766

[ref2] Benke, C., Autenrieth, L. K., Asselmann, E., & Pané-Farré, C. A. (2020). Lockdown, quarantine measures, and social distancing: Associations with depression, anxiety and distress at the beginning of the COVID-19 pandemic among adults from Germany. Psychiatry Research, 293, 113462. doi:10.1016/j.psychres.2020.113462. 32987222 PMC7500345

[ref3] Daly, M., Sutin, A. R., & Robinson, E. (2020). Longitudinal changes in mental health and the COVID-19 pandemic: Evidence from the UK household longitudinal study. Psychological Medicine, 1–10. doi: 10.1017/S0033291720004432. PMC7737138 33183370

[ref4] Debowska, A., Horeczy, B., Boduszek, D., & Dolinski, D. (2020). A repeated cross-sectional survey assessing university students' stress, depression, anxiety, and suicidality in the early stages of the COVID-19 pandemic in Poland. Psychological Medicine, 1–4. doi:10.1017/S003329172000392X. PMC7556906 33004087

[ref5] Jungmann, S. M., & Witthöft, M. (2020). Health anxiety, cyberchondria, and coping in the current COVID-19 pandemic: Which factors are related to coronavirus anxiety? Journal of Anxiety Disorders, 73, 102239. doi: 10.1016/j.janxdis.2020.102239. 32502806 PMC7239023

[ref6] Kroenke, K., Spitzer, R. L., & Williams, J. B. (2001). The PHQ-9: Validity of a brief depression severity measure. Journal of General Internal Medicine, 16(9), 606–613. doi:10.1046/j.1525-1497.2001.016009606.x. 11556941 PMC1495268

[ref7] Pierce, M., Hope, H., Ford, T., Hatch, S., Hotopf, M., John, A., … Abel, K. M. (2020). Mental health before and during the COVID-19 pandemic: A longitudinal probability sample survey of the UK population. The Lancet Psychiatry, 7(10), 883–892. doi:10.1016/S2215-0366(20)30308-4. 32707037 PMC7373389

[ref8] Prati, G., & Mancini, A. (2021). The psychological impact of COVID-19 pandemic lockdowns: A review and meta-analysis of longitudinal studies and natural experiments. Psychological Medicine, 1–11. doi: 10.1017/S0033291721000015. PMC7844215 33436130

[ref9] Spitzer, R. L., Kroenke, K., Williams, J. B. W., & Löwe, B. (2006). A brief measure for assessing generalized anxiety disorder: The GAD-7. Archives of Internal Medicine, 166(10), 1092–1097. doi:10.1001/archinte.166.10.1092. 16717171

